# Advances in Deep Learning-Based Medical Image Analysis

**DOI:** 10.34133/2021/8786793

**Published:** 2021-05-19

**Authors:** Xiaoqing Liu, Kunlun Gao, Bo Liu, Chengwei Pan, Kongming Liang, Lifeng Yan, Jiechao Ma, Fujin He, Shu Zhang, Siyuan Pan, Yizhou Yu

**Affiliations:** ^1^DeepWise AI Lab, BeijingChina; ^2^Shanghai Jiaotong University, Shanghai, China; ^3^The University of Hong Kong, Hong Kong

## Abstract

*Importance*. With the booming growth of artificial intelligence (AI), especially the recent advancements of deep learning, utilizing advanced deep learning-based methods for medical image analysis has become an active research area both in medical industry and academia. This paper reviewed the recent progress of deep learning research in medical image analysis and clinical applications. It also discussed the existing problems in the field and provided possible solutions and future directions.*Highlights*. This paper reviewed the advancement of convolutional neural network-based techniques in clinical applications. More specifically, state-of-the-art clinical applications include four major human body systems: the nervous system, the cardiovascular system, the digestive system, and the skeletal system. Overall, according to the best available evidence, deep learning models performed well in medical image analysis, but what cannot be ignored are the algorithms derived from small-scale medical datasets impeding the clinical applicability. Future direction could include federated learning, benchmark dataset collection, and utilizing domain subject knowledge as priors.*Conclusion*. Recent advanced deep learning technologies have achieved great success in medical image analysis with high accuracy, efficiency, stability, and scalability. Technological advancements that can alleviate the high demands on high-quality large-scale datasets could be one of the future developments in this area.

## 1. Introduction

With rapid developments of artificial intelligence (AI) technology, the use of AI technology to mine clinical data has become a major trend in medical industry [1]. Utilizing advanced AI algorithms for medical image analysis, one of the critical parts of clinical diagnosis and decision-making, has become an active research area both in industry and academia [2, 3]. Recent applications of deep leaning in medical image analysis involve various computer vision-related tasks such as classification, detection, segmentation, and registration. Among them, classification, detection, and segmentation are fundamental and most widely used tasks.

Although there exist a number of reviews on deep learning methods on medical image analysis [4-13], most of them emphasize either on general deep learning techniques or on specific clinical applications. The most comprehensive review paper is the work of Litjens et al. published in 2017 [12]. Deep learning is such a quickly evolving research field; numerous state-of-the-art works have been proposed since then. In this paper, we review the latest developments in the field of medical image analysis with comprehensive and representative clinical applications.

We briefly review the common medical imaging modalities as well as technologies for various specific tasks in medical image analysis including classification, detection, segmentation, and registration. We also give more detailed clinical applications with respect to different types of diseases and discuss the existing problems in the field and provide possible solutions and future research directions.

## 2. AI Technologies in Medical Image Analysis

**Figure 1:**
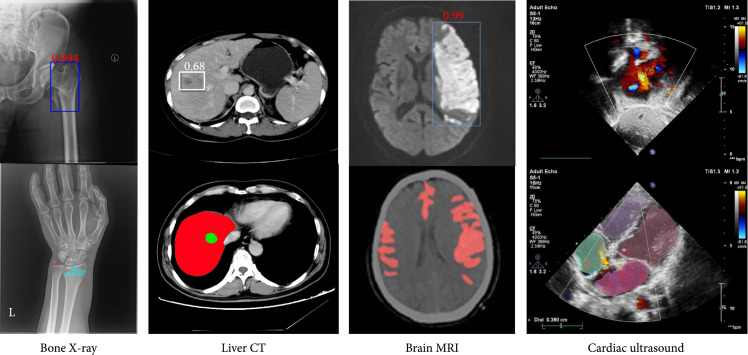
Examples of medical image modalities and their corresponding applications (original copy).

Different medical imaging modalities have their unique characteristics and different responses to human body structure and organ tissue and can be used in different clinical purposes. The commonly used image modalities for diagnostic analysis in clinic include projection imaging (such as X-ray imaging), computed tomography (CT), ultrasound imaging, and magnetic resonance imaging (MRI). MRI sequences include T1, T1-w, T2, T2-w, diffusion-weighted imaging (DWI), apparent diffusion coefficient (ADC), and fluid attenuation inversion recovery (FLAIR). Figure 1 demonstrates a few examples of medical image modalities and their corresponding clinical applications.

### 2.1. Image Classification for Medical Image Analysis

As a fundamental task in computer vision, image classification plays an essential role in computer-aided diagnosis. A straightforward use of image classification for medical image analysis is to classify an input image or a series of images as either containing one (or a few) of predefined diseases or free of diseases (i.e., healthy case) [14, 15]. Typical clinical applications of image classification tasks include skin disease identification in dermatology [16, 17], eye disease recognition in ophthalmology (such as diabetic retinopathy [18, 19], glaucoma [20], and corneal diseases [21]). Classification of pathological images for various cancers such as breast cancer [22] and brain cancer [23] also belongs to this area. 

**Figure 2:**
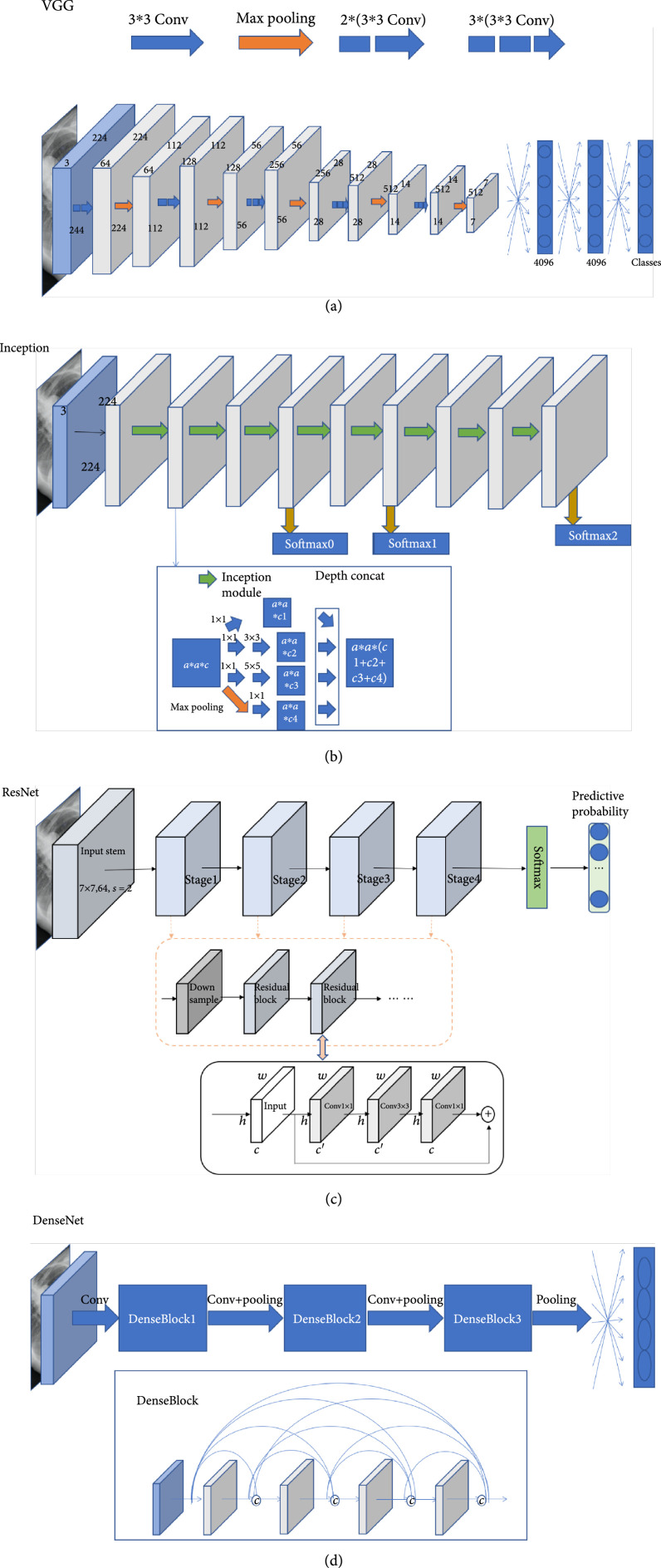
Examples of CNN-based classification networks (original copy).

Convolutional neural network (CNN) is the dominant classification framework for image analysis [24]. With the development of deep learning, the framework of CNN has continuously improved. AlexNet [25] was a pioneer convolutional neural network, which was composed of repeated convolutions, each followed by ReLU and max pooling operation with stride for downsampling. The proposed VGGNet [26] used 3×3 convolution kernels and 2×2 maximum pooling to simplify the structure of AlexNet and showed improved performance by simply increasing the number and depth of the network. Via combining and stacking 1×1, 3×3, and 5×5 convolution kernels and 3×3 pooling, the inception network [27] and its variants [28, 29] increased the width and the adaptability of the network. ResNet [30] and DenseNet [31] both used skip connections to relieve the gradient vanishing. SENet [32] proposed a squeeze-and-excitation module which enabled the model to pay more attention to the most informative channel features. The family of EfficientNet [33] applied AUTOML and a compound scaling method to uniformly scale the width, depth, and resolution of the network in a principled way, resulting in improved accuracy and efficiency. Figure 2 demonstrates some of the commonly used CNN-based classification network architectures. 

Besides the direct use for image classification, CNN-based networks can also be applied as the backbone models for other computer vision tasks, such as detection and segmentation.

To evaluate the algorithms of image classification, researchers use different evaluation metrics. Precision is the proportion of true positives in the identified images. The recall is the proportion of all positive samples in the test set that are correctly identified as positive samples. The accuracy rate is used to evaluate the global accuracy of a model. The $F_{1}$ score can be considered a harmonic average of the precision and the recall of the model, which takes both the precision and recall of the classification model into account. ROC (receiver operating characteristic) curve is usually used to evaluate the prediction effect of the binary classification model, and the kappa coefficient is a method to measure the accuracy of the model in multiclassification tasks.

(1)Precision=TPTP+FP,Recall=TPTP+FN,Accuracy=TP+TNn,F1=2∙Precision∙RecallPrecision+Recall.

Here, we denote TP as true positives, FP as false positives, FN as false negatives, TN as true negatives, and n as the number of the testing samples. 

### 2.2. Object Detection for Medical Image Analysis

Generally speaking, object detection algorithms include both identification and localization tasks. The identification task refers to judging whether objects belonging to certain classes appear in regions of interest (ROIs) whereas the localization task refers to localizing the position of the object in the image. In medical image analysis, detection is commonly aimed at detecting the earliest signs of abnormality in patients. Exemplar clinical applications of detection tasks include lung nodule detection in chest CT or X-ray images [34, 35], lesion detection on CT images [36, 37], or mammograms [38]. 

**Figure 3:**
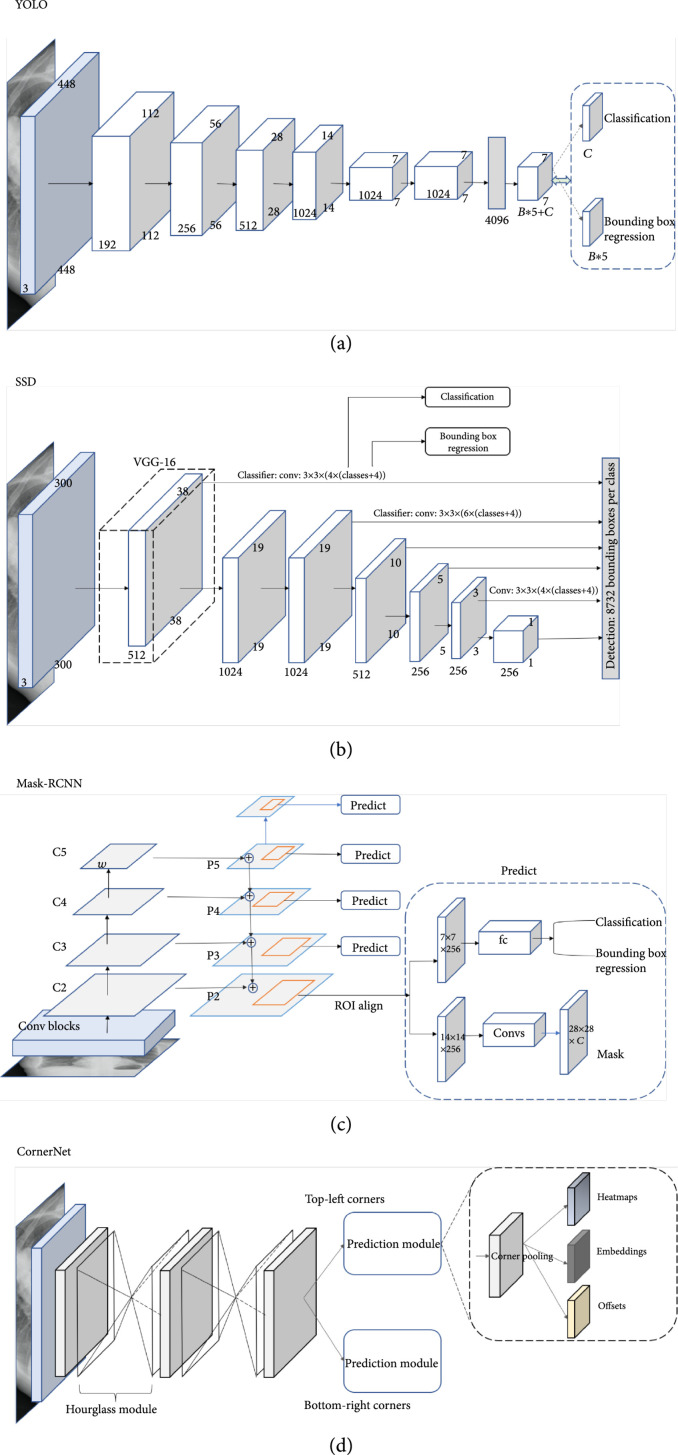
Examples of object detection frameworks (original copy).

Object detection algorithms can be categorized into two approaches, the anchor-based approach or anchor-free approach, where anchor-based algorithms can be further divided as single-stage algorithms or two/multistage algorithms. In general, single-stage algorithms are computationally efficient whereas two/multistage algorithms have better detection performance. The family of YOLO [39] and the single-shot multibox detector (SSD) [40] are two classic and widely used single-stage detectors with simple model architectures. As shown in Figures 3(a) and 3(b), both architectures are based on feed-forward convolutional networks producing a fixed number of bounding boxes and their corresponding scores for the presence of object instances of given classes in the boxes. A nonmaximum suppression step is applied to generate the final predictions. Different from YOLO which works on a single-scale feature map, the SSD utilizes multiscale feature maps, thereby producing better detection performance. Two-stage frameworks generate a set of ROIs and classify each of them through a network. The Faster-RCNN framework [41] and its descendant Mask-RCNN [42] are the most popular two-stage frameworks. As shown in Figure 3(c), the Faster/Mask-RCNN first generates object proposals through a region proposal network (RPN) and then classifies those generated proposals. The major difference between the Faster-RCNN and the Mask-RCNN is that the Mask-RCNN has an instance segmentation branch. Recently, there is a research trend on developing anchor-free algorithms. CornerNet [43] is one of the popular ones. As illustrated in Figure 3(d), CornerNet is a single convolutional neural network which eliminates the use of anchor boxes via utilizing paired key points where an object bounding box is indicated by the top-left corner and the bottom-right corner. 

There are two main metrics to evaluate the performance of detection methods: the mean average precision (mAP) and the false positive per image (FP/I @ recall). mAP is used to calculate the average of all average precisions (APs) of all categories. FP/I @ recall rate is a measure of false positive (FP) of each image under a certain recall rate which takes into account the balance between false positives and the missing rate.

### 2.3. Segmentation for Medical Image Analysis

Image segmentation is a pixel labeling problem, which partitions an image into regions with similar properties. For medical image analysis, segmentation is aimed at determining the contour of an organ or anatomical structure in images. Segmentation tasks in clinical applications include segmenting a variety of organs, organ structures (such as the whole heart [44] and pancreas [45]), tumors, and lesions (such as the liver and liver tumor [46]) across different medical imaging modalities. 

**Figure 4:**
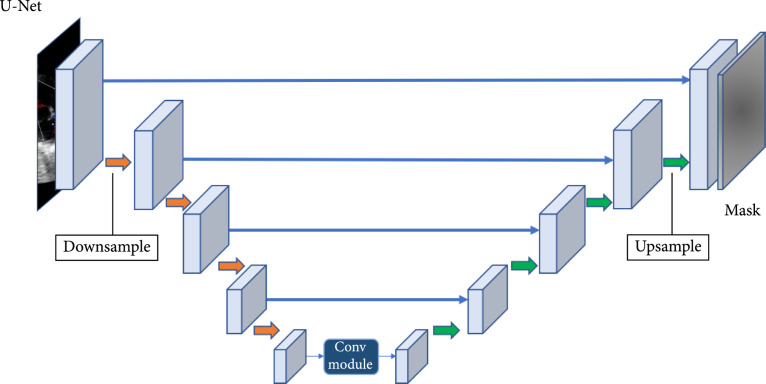
Examples of image segmentation frameworks (original copy).

Since the fully convolutional neural network (FCN) [47] has been proposed, image segmentation has achieved great success. FCN was the first CNN which turned the classification task to dense segmentation task with in-network upsampling and a pixelwise loss. Through a skip architecture, it combined coarse, semantic, and local information to dense prediction. Medical image segmentation methods can be divided into two categories: the 2D methods and the 3D methods according to the input data dimension. The U-Net architecture [48] is the most popular FCN for medical image segmentation. As shown in Figure 4, U-Net consists of a contracting path (the downsample side) and an expansive path (the upsample side). The contracting path follows the typical CNN architecture. It consists of the repeated application of convolutions, each followed by ReLU and max pooling operation with stride for downsampling. At each downsampling step, it also doubles the number of feature channels. Each step in the expansive path is composed of feature map upsampling followed by deconvolution that halves the number of feature channels; a concatenation with the correspondingly cropped feature map from the contracting path is also applied. Variants of U-Net-based architectures have been proposed. Isensee et al. [49] proposed a general framework called nnU-Net (No new U-Net) for medical image segmentation, which applied a dataset fingerprint (representing the key properties of the dataset) and a pipeline fingerprint (representing the key design of the algorithms) to systematically optimize the segmentation task via formulating a set of heuristic rules from domain knowledge. The nnU-Net achieved state-of-the-art performance on 19 different datasets with 49 segmentation tasks across a variety of organs, organ structures, tumors, and lesions in a number of imaging modalities (such as CT, MRI). 

Dice similarity coefficient and intersection over union (IOU) are the two major evaluation metrics to evaluate the performance of segmentation methods, and they are defined as follows:

(2)Dice=2×TP2×TP+FP+FN,IOU=TPTP+FP+FN,

where TP, FP, and FN denote true positive, false positive, and false negative, respectively. 

### 2.4. Image Registration for Medical Image Analysis

Image registration, also known as image warping or image fusion, is a process of aligning two or more images. The goal of medical image registration is aimed at establishing optimal correspondence within images acquired at different times (for longitudinal studies), by different imaging modalities (such as CT, MRI), across different patients (for intersubject studies), or from distinct viewpoints. Image registration plays a crucial preprocessing step in many clinical applications including computer-aided intervention and treatment planning [50], image-guided/assisted surgery or simulation [51], and fusion of anatomical images (e.g., CT or MRI images) with functional images (such as positron emission tomography, single-photon emission computed tomography, or functional MRI) for disease diagnosis and monitoring [52]. 

Depending on different points of view, image registration methodologies can be categorized differently. For instance, image registration methods can be classified as monomodal or multimodal based on imaging modalities involved. From the nature of geometric transformation, methods can also be categorized as rigid or nonrigid classes. By data dimensionality, registration methods can be classified as 2D/2D, 3D/3D, 2D/3D, etc., and from similarity measure point of view, registration can be categorized as feature-based or intensity-based groups. Previously, image registration has been extensively explored as an optimization problem whose aim is to search the best geometric transformation iteratively through optimizing a similarity measure such as sum of squared differences (SSD), mutual information (MI), and cross-correlation (CC). Ever since the beginning of the deep learning renaissance, various deep learning-based registration methods have been proposed and achieved the state-of-the-art performance [53]. 

Yang et al. [54] proposed a fully supervised deep learning method to align 2D/3D intersubject brain MR in a single step via a U-Net-like FCN. Jun et al. [55] also applied a CNN to perform deformable registration of abdominal MR images to compensate respiration deformation. Despite the success of supervised learning-based methods, the nature of acquisition of reliable ground truth remains significantly challenging. Weakly supervised and/or unsupervised methods can effectively alleviate the issue of lack of training datasets with ground truth. Li and Fan [56] trained an FCN to perform deformable 3D brain MR images using self-supervision. Inspired by the spatial transfer network (STN) [57], Kuang et al. [58] applied a STN-based CNN to perform deformable registration of MRI T1-W brain volumes. 

Recently, Generative Adversarial Network- (GAN-) and Reinforcement Learning- (RL-) based methods have also motivated great attentions. Yan et al. [59] performed a rigid registration of 3D MR and ultrasound images. In their work, the generator was trained to estimate rigid transformation where the discriminator was used to distinguish between images that were aligned by ground-truth transformations or by predicted ones. Kreb et al. [60] applied a RL method to perform the nonrigid deformable registration of 2D/3D prostate MRI images where they utilized a low-resolution deformation model for registration and a fuzzy action control to influence the action selection. 

For performance evaluation, Dice coefficient and mean square error (MSE) are two major evaluation metrics. Target registration error (TRE) can also be applied if landmark correspondence can be acquired.

## 3. Clinical Applications

In this section, we review state-of-the-art clinical applications in four major systems of the human body involving the nervous system, the cardiovascular system, the digestive system, and the skeletal system. To be more specific, AI algorithms on medical image diagnostic analysis for the following representative diseases including brain diseases, cardiac diseases, and liver diseases, as well as orthopedic trauma, are discussed.

### 3.1. Brain

In this section, we discuss three most critical brain diseases, namely, stroke, intracranial hemorrhage, and intracranial aneurysm.

#### 3.1.1. Stroke

Stroke is one of the leading causes of death and disability worldwide and imposes an enormous burden for health care systems [61]. Accurate and automatic segmentation of stroke lesions can provide insightful information for neurologists.

Recent studies have presented tremendous ability in stroke lesion segmentation. Chen et al. [62] used DWI images as input to segment acute ischemic lesions and achieved an average Dice score of 0.67. Clèrigues et al. [63] proposed a deep learning methodology for acute and subacute stroke lesion segmentation using multimodal MRI images, and the Dice scores of the two segmentation tasks were 0.84 and 0.59, respectively. Liu et al. [64] used a U-shaped network (Res-CNN) to automatically segment acute ischemic stroke lesions from multimodality MRIs, and the average Dice coefficient was 0.742. Zhao et al. [65] proposed a semisupervised learning method using the weakly labeled subjects to detect the suspicious acute ischemic stroke lesions and achieved a mean Dice coefficient of 0.642. Compared to using MRI, a 2D patch-based deep learning approach was proposed to segment the acute stroke lesion core from CT perfusion images [66], and the average Dice coefficient was 0.49.

#### 3.1.2. Intracranial Hemorrhage

Recent studies have also shown great promise in automated detection of intracranial hemorrhage and its subtypes. Chilamkurthy et al. [67] achieved an AUC of 0.92 for detecting intracranial hemorrhage based on a publicly available dataset called CQ500 consisting of 313,318 head CT scans from 20 centers. They use the original clinical radiology report and consensus of three independent radiologists as the gold standard to evaluate their method. Ye et al. [68] proposed a novel three-dimensional (3D) joint convolutional and recurrent neural network (CNN-RNN) for the detection of intracranial hemorrhage. They developed and evaluated their method on a total of 2,836 subjects (ICH/normal, 1,836/1,000) from three institutions. Their algorithm achieved an AUC of 0.94 for intraparenchymal, 0.93 for intraventricular, 0.96 for subdural, 0.94 for extradural, and 0.89 for subarachnoid for the subtype classification task. Ker et al. [69] proposed to apply an image thresholding in the preprocessing step to improve the classification F1 score from 0.919 to 0.952 for their 3D CNN-based acute brain hemorrhage diagnosis. Singh et al. [70] also proposed an image preprocessing method to improve the 3D CNN-based acute brain hemorrhage detection via normalizing 3D volumetric scans using intensity profile. Their experimental results demonstrated the best F1 scores of 0.96, 0.93, 0.98, and 0.99, respectively, for four types of acute brain hemorrhages (i.e., subarachnoid, intraparenchymal, subdural, and intraventricular) on the CQ500 dataset [67].

#### 3.1.3. Intracranial Aneurysm

Intracranial aneurysm is a common life-threatening disease usually caused by trauma, vascular disease, or congenital development with a prevalence of 3.2% in the population [71]. Rupture of an intracranial aneurysm is a serious incident with high mortality and morbidity rates [72]. As such, the accurate detection of intracranial aneurysms is also important. Computed tomography angiography (CTA) and magnetic resonance angiography (MRA) are noninvasive methods and widely used for the diagnosis and presurgical planning of intracranial aneurysms [73]. Nakao et al. [74] used a CNN classifier to predict whether each voxel was inside or outside aneurysms by inputting MIP images generated from a volume of interest around the voxel. They detected 94.2% of aneurysms with 2.9 false positives per case. Stember et al. [75] employed a CNN based on U-Net architecture to detect aneurysms on MIP images and then to derive aneurysm size. Sichtermann et al. [76] established a system based on an open-source neural network named DeepMedic for the detection of intracranial aneurysms from 3D TOF-MRA data. Ueda et al. [77] adopted ResNet for the detection of aneurysms from MRA images and reached a sensitivity of 91% and 93% for the internal and external test datasets, respectively. Allison et al. [78] proposed a segmentation model called HeadXNet to segment aneurysms on CTA images. Recently, Shi et al. [79] proposed a 3D patch-based deep learning model for detecting intracranial aneurysm in CTA images. The proposed model utilized both spatial and channel attentions within a residual-based encoder-decoder architecture. Experimental results on multicohorta studies proofed the clinical applicability.

### 3.2. Cardiac/Heart

Echocardiography, CT, and MRI are commonly used medical imaging modalities for noninvasive assessment of the function and structure of the cardiovascular system. Automatic analysis of images from the above modalities can help physicians study the structure and function of heart muscle, find the cause of a patient’s heart failure, identify potential tissue damages, and so on.

#### 3.2.1. Identification of Standard Scan Planes

Identification of standard scan planes is an important step in clinical echocardiogram interpretation since many cardiac diseases are diagnosed based on standard scan planes. Zhang et al. [80] built a fully automated, scalable, analysis pipeline for echocardiogram interpretation, including view identification, cardiac chamber segmentation, quantification of structure and function, and disease detection. They trained a 13-layer CNN on 14,035 echocardiograms spanning on a 10-year period for identification of 23 viewpoints and trained a cardiac chamber segmentation network across 5 common standard scan planes. Then, the segmentation output was used to quantify chamber volumes and LV mass, determine ejection fraction, and facilitate automated determination of longitudinal strain through speckle tracking. Howard et al. [81] trained a two-stream network on over 8,000 echocardiographic videos for 14 different scan plane identification, which contained a time-distributed network to get spatial feature and a temporal network to get optical flow feature of moving objects between frames. Experiments showed that the proposed method can halve the error rate for video scan plane classification, and the types of misclassification the method made were very similar to differences of opinion between human experts.

#### 3.2.2. Segmentation of Cardiac Structures

Vigneault et al. [82] presented a novel deep CNN architecture called *Ω*-Net for fully automatic whole-heart segmentation. The network was trained end to end from scratch to segment five foreground classes (the four cardiac chambers plus the LV myocardium) in three views (SA, 4C, and 2C) with data acquired from both 1.5-T and 3-T magnets as part of a multicenter trial involving 10 institutions. Xiong et al. [83] developed a 16-layer CNN model called AtriaNet to automatically segment the left atrial (LA) epicardium and endocardium. AtriaNet consists of a multiscaled dual-pathway architecture with two different sizes of input patches centered on the same region that captures both the local arterial tissue and geometry and the global positional information of LA. Benchmarking experiments showed that AtriaNet had outperformed the state-of-the-art CNNs, with a Dice score of 0.940 and 0.942 for the LA epicardium and endocardium at the time. Moccia et al. [84] modified and trained the ENet, a fully convolutional neural network, to provide scar-tissue segmentation in the left ventricle. Bai et al. [85] proposed an image sequence segmentation algorithm by combining a fully convolutional network with a recurrent neural network, which incorporated both spatial and temporal information into the segmentation task. The proposed method achieved an average Dice metric of 0.960 for the ascending aorta and 0.953 for the descending aorta. Morris et al. [86] developed a novel pipeline that paired MRI/CT data that were placed into separate image channels to train a 3D neural network using the entire 3D image for sensitive cardiac substructure segmentation. The paired MR/CT multichannel data inputs yielded robust segmentations on noncontrast CT inputs, and data augmentation and 3D Conditional Random Field (CRF) postprocessing improved deep learning contour agreement with ground truth.

#### 3.2.3. Coronary Artery Segmentation

Shen et al. [87] proposed a joint framework for coronary CTA segmentation based on deep learning and traditional-level set method. A 3D FCN was used to learn the 3D semantic features of coronary arteries. Moreover, an attention gate was added to the entire network, aiming to enhance the vessels and suppress irrelevant regions. The output of 3D FCN with the attention gate was optimized by the level set to smooth the boundary to better fit the ground-truth segmentation. The coronary CTA dataset used in this work consisted of 11,200 CTA images from 70 groups of patients, of which 20 groups of patients were used as a test set. The proposed algorithm provided significantly better segmentation results than vanilla 3D FCN intuitively and quantitatively. He et al. [88] developed a novel blood vessel centerline extraction framework utilizing a hybrid representation learning approach. The main idea was to use CNNs to learn local appearances of vessels in image crops while using another point-cloud network to learn the global geometry of vessels in the entire image. This combination resulted in an efficient, fully automatic, and template-free approach to centerline extraction from 3D images. The proposed approach was validated on CTA datasets and demonstrated its superior performance compared to both traditional and CNN-based baselines.

#### 3.2.4. Coronary Artery Calcium and Plaque Detection

Zhang et al. [89] established an end-to-end learning framework for artery-specific coronary calcification identification in noncontrast cardiac CT, which can directly yield accurate results based on given CT scans in the testing process. In this framework, the intraslice calcification features were collected by a 2D U-DenseNet, which was the combination of DenseNet and U-Net. While those lesions spanned multiple adjacent slices, authors performed 3D U-Net extraction to the interslice calcification features, and the joint semantic features of 2D and 3D modules were beneficial to artery-specific calcification identification. The proposed method was validated on 169 noncontrast cardiac CT exams collected from two centers by cross-validation and achieved a sensitivity of 0.905, a PPV of 0.966 for calcification number, a sensitivity of 0.933, a PPV of 0.960, and a $F_{1}$ score of 0.946 for calcification volume, respectively. Liu et al. [90] proposed a vessel-focused 3D convolutional network for automatic segmentation of artery plaque including three subtypes: calcified plaques, noncalcified plaques, and mixed calcified plaques. They first extracted the coronary arteries from the CT volumes and then reformed the artery segments into straightened volumes. Finally, they employed a 3D vessel-focused convolutional neural network for plaque segmentation. This proposed method was trained and tested on a dataset of multiphase CCTA volumes of 25 patients. The proposed method achieved Dice scores of 0.83, 0.73, and 0.68 for calcified plaques, noncalcified plaques, and mixed calcified plaques, respectively, on the test set, which showed a potential value for clinical application.

### 3.3. Liver

CT and MRI are widely used for the early detection, diagnosis, and treatment of liver diseases. Automatic segmentation of the liver and/or liver lesion with CT or MRI is of great importance in radiotherapy planning, liver transplantation planning, and so on.

#### 3.3.1. Liver Lesion Detection and Segmentation

Vorontsov et al. used deep CNNs to detect and segment liver tumors [91]. For lesion sizes smaller than 10 mm (n=30), 10-20 mm (n=35), and larger than 20 mm (n=40), the detection sensitivities of the method were 10%, 71%, and 85%; positive predictive values were 25%, 83%, and 94%; and dice similarity coefficients were 0.14, 0.53, and 0.68. Wang et al. proposed an attention network by using an extra network to gather information from continuous slices for lesion segmentation [92]. This method had a Dice per case score of 74.1% on LiTS test dataset. In order to improve the performance on small lesions, modified U-Net (mU-Net) is proposed by Seo et al. which obtained a Dice score of 89.72% on validation set for liver tumor segmentation [93]. An edge enhanced network was proposed by Tang et al. [94] for liver tumor segmentation with a Dice per case score of 74.8% on LiTS test dataset.

#### 3.3.2. Liver Lesion Classification

Unlike liver lesion segmentation or detection, there are few works about lesion classification, as there is no public dataset about lesion classification, and it is difficult to collect enough data. A liver tumor classification system trained with 1,210 patients and validated in 201 patients based on deep learning was proposed by Zhen et al. [95]. The system can distinguish malignant from benign liver tumors with an AUC score of 94.6% using only unenhanced images, and the performance can be improved a lot with clinical information.

#### 3.3.3. Liver Fibrosis Staging

Liver fibrosis staging is important for the prevention and treatment of chronic liver disease. Although the amount of the works based on deep learning for liver fibrosis staging is few, these methods have shown their capability for this task. Liu et al. proposed a method using CNNs and SVM to classify the capsules on ultrasound images to get the stage score, and this method had a classification AUC score of 97.03% [96]. Yasaka et al. proposed two deep CNNs models to obtain stage scores, respectively, from CT [97] and MRI [98] images, achieving AUC scores of 0.73-0.76 and 0.84-0.85, respectively. Choi et al. trained a model based on deep learning using 7,491 patients and validated on 891 patients, and the AUC score on the validation dataset was 0.95-0.97 [99]. Recently, a model based on multimodal ultrasound images received an AUC score of 0.93-0.95 [100] which used transfer learning to improve the classification performance.

#### 3.3.4. Other Liver Disease

Prediction of microvascular invasion (MVI) before surgery is valuable for liver cancer patients’ treatment planning since MVI is an adverse prognostic factor for these patients [101]. Men et al. proposed 3D CNNs with LSTM to predict MVI on enhanced MRI images receiving an AUC score of 89% [102]. Jiang et al. [103] also reported a 3D CNN-based one with enhanced CT images achieving an AUC score of 90.6%.

### 3.4. Bone

Bone fracture, also called orthopedic trauma, is a relatively common disease. Bone fracture recognition in X-ray images has become a promising research direction since 2017 with the development of deep learning technology. In general, there are two main approaches for bone fracture recognition, namely, the classification-based approach and the object detection-based approach.

#### 3.4.1. Classification-Based Approach

For the classification-based approach, researchers usually use the labels of “no fracture” and “fracture” for the whole image. The pioneer and dedicated work of the classification pipeline was from Olczak et al. [104]. By adopting the VGGNet as the backbone of the classification pipeline, they trained the model on 256,000 well-labeled images of the wrists, hands, and ankles for recognizing fractures. With a large amount of validating data, the model set a strong and credible baseline of the accuracy of 83%. Urakawa et al. [105] used the same network architecture as Olczak et al.’s in classifying intertrochanteric hip fractures on 3,346 radiographs. The results have shown a 95.5% accuracy whereas an accuracy of orthopedic surgeons was reported at 92.2%. Gale et al. [106] extracted 53,000 clinical X-rays to get an area under the ROC curve of 0.994 whereas Krogue et al. [107] labeled 3,034 images to get an area under the curve of 0.973. They both applied DenseNet into the classification task on hip fracture radiographs.

#### 3.4.2. Object Detection-Based Approach

The object detection-based approach is aimed at localizing the fracture locations in the images. Gan et al. [108] trained a Faster R-CNN model to locate the area of wrist fracture; then, they sent the ROI to an inception framework for classification. The AUC score achieved 0.96 overpassing radiologists’ performance by 9% in accuracy on a set of 2,340 anteroposterior wrist radiographs. Thian et al. [109] employed the same Faster R-CNN architecture and also ran the model on wrist radiographs with a larger volume of the dataset of 7,356 images. The result had an indistinctive AUC score of 0.957. Still on wrist radiographs, using the idea of semantic segmentation, Lindsey et al. [110] adopted an extension of U-Net to predict a heat map probability of fractures for each image pixel. Even using 135,409 wrist radiographs, the article only reported an average clinician sensitivity of 91.5% and specificity of 93.9% aided with a trained model, which seemed to be inferior to the above research. Wu et al. [111] proposed an end-to-end multidomain facture detection network which treated each body part as a domain. The proposed network was composed of two subnetworks, namely, a domain classification network for predicting the domain type of an image and a fracture detection network for detecting fractures on X-ray images of different domains. By constructing feature enhancement modules and multifeature-enhanced r-CNN, the proposed network extracted more representative features for each domain. Experimental results on real-clinical data demonstrated the effectiveness with the best F-score on all the domains over existing Faster R-CNN-based state-of-the-art methods. Recently, Wu et al. [112] proposed a novel feature ambiguity mitigation model to improve the bone fracture detection on X-ray radiographs. A total of 9,040 radiographic images for various body parts including the hand, wrist, elbow, shoulder, pelvic, knee, ankle, and foot were studied. Experimental results demonstrated performance improvements in all body parts.

## 4. Challenges and Future Directions

Although deep learning models have achieved great success in medical image analysis, small-scale medical datasets are still the main bottleneck in this field. Inspired by the idea of transfer learning technique, one possible way is to do domain transfer which adapts a model trained on natural images to medical image applications or from one image modality to another. Another possible way is to apply federated learning [113] by which training can be performed among multiple data centers collaboratively. In addition, researchers have also begun to collect benchmark datasets for various medical image analysis purposes. Table 1 summarized examples of the publicly available datasets.

**Table 1:** Publicly available Benchmark datasets.

Dataset name	Organ/modalities	Image size	No. classes	No. of cases	Tasks	Resources
LIDC-IDRI	Lung/CT	133×512×512	3	1018	Lung nodules	[114]
LUNA	Lung/CT	133×512×512	1	888	Lung nodules	[115]
DDSM	Breast/mammography	—	3	2,500	Breast mass	[116]
DeepLesion	Diversity CT	—	3+	4427	Lung nodules, liver tumors, lymph nodes	[117]
LiTS	Liver/CT	432×512×512	2	131	Liver, liver tumors	[118]
Brain tumor	Brain/MRI	138×169×138	3	484	Edema, tumor, necrosis	[119]
Heart	Heart/MRI	115×320×232	1	20	Left ventricle	[119]
Prostate	Prostate/MRI	20×320×319	2	32	Peripheral and transition zone	[119]
Pancreas	Pancreas/CT	93×512×512	2	282	Pancreas, pancreas cancer	[119]
Spleen	Spleen/CT	90×512×512	1	41	Spleen	[119]
Colon	Colon/CT	95×512×512	1	126	Colon cancer	[119]

Class imbalance is another major problem of medical image analysis. A number of researches on novel loss function design, such as focal loss [120], grading loss [121], contrastive loss [122], and triplet loss [123], have been proposed to tackle this problem. Making use of domain subject knowledge is another direction. For instance, Jiménez-Sánchez et al. [124] proposed a curriculum learning method to classify proximal femoral fractures in X-ray images, whose core idea is to control the sampling weight of samples in the training process based on a priori knowledge. Chen et al. [125] also proposed a novel pelvic fracture detection framework based on bilaterally symmetric structure assumption.

## 5. Conclusion

The rise of advanced deep learning methods has enabled great success in medical image analysis with high accuracy, efficiency, stability, and scalability. In this paper, we reviewed the recent progress of CNN-based deep learning techniques in clinical applications including image classification, object detection, segmentation, and registration. More detailed image analysis-based diagnostic applications in four major systems of the human body involving the nervous system, the cardiovascular system, the digestive system, and the skeletal system were reviewed. To be more specific, state-of-the-art works for different diseases including brain diseases, cardiac diseases, and liver diseases, as well as orthopedic trauma, are discussed. This paper also described the existing problems in the field and provided possible solutions and future research directions.
